# Improving the Photocurrent in Quantum-Dot-Sensitized Solar Cells by Employing Alloy Pb*_x_*Cd_1−*x*_S Quantum Dots as Photosensitizers

**DOI:** 10.3390/nano6060097

**Published:** 2016-05-27

**Authors:** Chunze Yuan, Lin Li, Jing Huang, Zhijun Ning, Licheng Sun, Hans Ågren

**Affiliations:** 1 Department of Theoretical Chemistry and Biology, School of Biotechnology, Royal Institute of Technology, 10691 Stockholm, Sweden; chunze@stanford.edu (C.Y.); jinghuang@theochem.kth.se (J.H.); zhijunning@gmail.com (Z.N.); 2 Center of Molecular Devices, Department of Chemistry, School of Chemical Science and Engineering, Royal Institute of Technology, 10044 Stockholm, Sweden; lin3@kth.se

**Keywords:** quantum dot-sensitized solar cells, photocurrent, alloy, PbS

## Abstract

Ternary alloy Pb*_x_*Cd_1−*x*_S quantum dots (QDs) were explored as photosensitizers for quantum-dot-sensitized solar cells (QDSCs). Alloy Pb*_x_*Cd_1−*x*_S QDs (Pb_0.54_Cd_0.46_S, Pb_0.31_Cd_0.69_S, and Pb_0.24_Cd_0.76_S) were found to substantially improve the photocurrent of the solar cells compared to the single CdS or PbS QDs. Moreover, it was found that the photocurrent increases and the photovoltage decreases when the ratio of Pb in Pb_x_Cd_1−x_S is increased. Without surface protecting layer deposition, the highest short-circuit current density reaches 20 mA/cm^2^ under simulated AM 1.5 illumination (100 mW/cm^2^). After an additional CdS coating layer was deposited onto the Pb*_x_*Cd_1−*x*_S electrode, the photovoltaic performance further improved, with a photocurrent of 22.6 mA/cm^2^ and an efficiency of 3.2%.

## 1. Introduction

Quantum-dot-sensitized solar cells (QDSCs), which are similar to dye-sensitized solar cells (DSCs) in terms of configuration and working mechanism [1,2], have been intensively investigated in the last decade due to the excellent properties of quantum dots (QDs), *i.e.*, size-dependent spectral tunability, high molar extinction coefficients, and low fabrication cost [3,4,5,6,7]. Especially, QD-based solar cells have shown promising conversion efficiency in the infrared region, where light cannot be effectively harvested by DSCs. In addition, the unique multi-electron generation effect of QDs proposes the theoretically maximum efficiency of QDSCs to be as high as 44% [8]. Therefore, QDSCs have been studied as a promising alternative or supplement to DSCs [9,10,11]. However, this efficiency is still far from the theoretically highest efficiency [12,13]. To enable the photovoltaic performance of QDSCs to compete with DSCs or other type solar cells, many efforts have been made, e.g., exploring new kinds of QDs with better light harvesting capability, electron injection efficiency, electrolyte and electrode materials in order to alleviate the series resistance between the electrolyte and cathode, as well as the carrier recombination between anode and electrolyte [14,15,16,17,18,19]. The extension of the light-harvesting region of the photosensitizers to the near-infrared (NIR) region has received more and more attention owing to its critical role to further improve the general solar cell performance [20,21,22]. Thus, although currently silicon solar cells can harvest light well in the visible region, there is lack of high-performance materials that can efficiently harvest infrared light.

So far, few types of QDs with absorption spectra stretching down to the NIR area have been proven to be effective for QDSCs. The broadening of the absorption spectrum has motivated the development of type-II core/shell-structured QDs, the bandgap of which is the difference between the conduction band (CB) edge of the component with deeper conduction band and the valence band (VB) edge of the component with a smaller VB [23,24]. QDs alloyed with multiple elements serve as another possible approach to broaden the absorption spectrum since they may present lower bandgaps than the corresponding binary QDs by the effect of optical bowing [25]. Another popular approach is to apply some traditional QDs with narrow bandgaps, such as PbS, PbSe, and Ag_2_S [26,27,28]. PbS QDs have been intensely studied for the solar cell application due to the narrow bandgap (0.41 eV for bulk PbS) and potential multiple exciton collection [29,30,31,32,33,34,35]. The Bohr radius of PbS is as large as 18 nm [34], which makes it possible to tune its bandgap in a wide range by modifying the size of QDs.

PbS QDs have been successfully applied in heterojunction solar cells, showing remarkable conversion efficiency up to 10% [36,37,38,39,40,41]. However, PbS QDSCs show much lower device performance due to the low CB level, heavy charge recombination, and low stability with the presently available liquid electrolytes [30,33]. Coating of CdS or CdPbS shells onto PbS QDs have been explored, showing increased device performance compared with PbS only QDs, as the recombination and corrosion with the electrolyte are then inhibited [30,42]. Alloy QDs have been proposed as a good strategy for improving the optical and electronic properties of QDs by controlling the proportion of their component elements [25,43].

Solution phase *in-situ* ion adsorption by the method of successive ionic-layer adsorption and reaction (SILAR) [5,44] has been widely used for QDSCs and shows promising device performance, due to its convenient fabrication process. SILAR-adsorbed PbS QDs has been explored for QDSCs, albeit giving quite low device performance. Mixed Cd and Pb ions by using Pb(NO_3_)_2_ as lead salts for alloy Pb*_x_*Cd_1−*x*_S adsorption have been used; however, the device performance remains still low. It still remains unclear how the ratio of Pb:Cd of Pb*_x_*Cd_1−*x*_S QDs can influence the photovoltaic performance of QDSCs [45]. Since their bandgaps and CB levels are in between those of PbS and CdS [42], Pb*_x_*Cd_1−*x*_S QDs may present a higher CB edge than PbS and a wider absorption spectrum than CdS. By tuning the Pb:Cd ratio of Pb*_x_*Cd_1−*x*_S, the bandgap and CB level of the alloy QDs can be adjusted, which should affect the light-harvesting ability, carrier injection, and carrier recombination in the QDSCs. On the other hand, it has been shown that the addition of a certain amount of Cd in PbS QDs can significantly limit the carrier recombination by reducing the trap density without affecting the carrier extraction, which brings much improved device performance [36]. Herein, in this report, we systematically study the effect of the ratio of Pb:Cd. This ratio is varied by controlling the Pb^2+^ ion concentration in the cationic precursor solutions, which is used to prepare three kinds of Pb*_x_*Cd_1−*x*_S QDs by the SILAR process.

## 2. Results and Discussion

**Figure 1:**
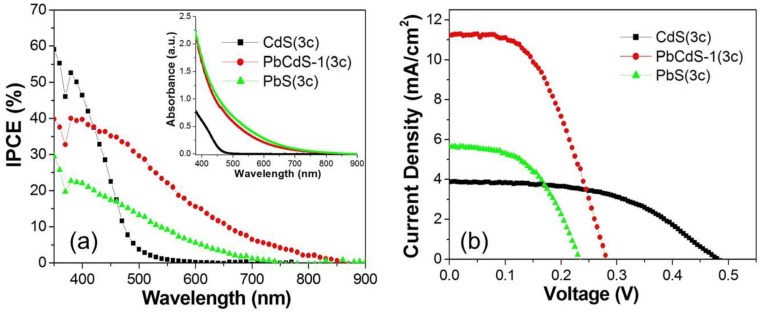
Incident photon to current efficiency (IPCE) spectra (**a**) and current–voltage (I–V) curves (**b**) of quantum-dot-sensitized solar cells (QDSCs) employing three successive ionic-layer adsorption and reaction (SILAR) cycles of CdS, PbS, and PbCdS-1 as photosensitizers. Inset of (**a**) is the absorption spectra of three-SILAR-cycle QD-sensitized TiO_2_ films. “3C” means three SILAR cycles, and the bare TiO_2_ film was used as blank.

Firstly, a kind of Pb*_x_*Cd_1−*x*_S QDs, termed PbCdS-1, was investigated as a photosensitizer compared to the CdS and PbS QDs. PbCdS-1 was prepared by a mixed precursor solution containing 0.004 M PbCl_2_ and 0.1 M Cd(NO_3_)_2_. More details are given in the experimental section. The CdS, PbS, and PbCdS-1 were deposited on the TiO_2_ electrodes by the SILAR process. The absorption spectra of the CdS, PbS, and PbCdS-1 QD-sensitized TiO_2_ electrodes after three SILAR cycles are shown in the inset of Figure 1a. The CdS-sensitized electrode shows the narrowest absorption range (<500 nm), due to its wide bandgap, and the lowest absorbance intensity. In contrast, the PbS electrode shows the highest absorbance intensity and the widest absorption range over 800 nm, due to the considerably narrow bandgap and high molar extinction coefficient. For the PbCdS-1 electrode, the absorbance intensity is slightly lower than for PbS. The absorption performance of the PbCdS-1 QDs is similar to that of the PbS QDs, indicating that PbCdS-1 can harvest similar photon energies as PbS.

Figure 1a shows the incident photon to current efficiency (IPCE) spectra of QDSCs employing CdS, PbS, and PbCdS-1 with three SILAR cycles as photosensitizers. From the absorption spectra, the sizes of three-cycle CdS, PbS, and PbCdS-1 can be estimated to be ~3 nm, ~2 nm, and 2–4 nm, respectively [45,46,47,48]. The CdS QDSCs show the highest IPCE and narrowest region (only up to 550 nm). The highest IPCE is due to the high CB energy level of CdS, which leads to a high driving force for electron injection from CB of CdS to TiO_2_ [5]. The narrow photocurrent response range of the CdS cell gives an agreement with its absorption spectrum, which is limited by the wide bandgap of CdS. In contrast, the PbS QDSCs present a narrower IPCE range (<700 nm) than their absorption spectra (up to 800 nm), possibly due to the non-radiative decay through deep energy level traps or low CB energy levels. For PbCdS-1 QDSCs, it is clear that the range of photocurrent response extends up to 800 nm, which is in concordance with its absorption spectrum. In addition, Cd cation may be bound to the unpassivated S in PbS, thus removing valence-band-associated trap states [36]. The possible reasons of the much improved efficiency in the infrared region are the higher CB edge and the much reduced trap density of states for the alloy QDs. Compared to PbS, a remarkably improved overall photocurrent response was observed with a maximum IPCE close to 40%. The IPCE is almost double to that of PbS.

Figure 1b shows the current–voltage (I–V) curves of QDSCs employing CdS, PbS, and PbCdS-1 with three SILAR cycles under simulated one sun illumination (AM 1.5, 100 mW/cm^2^). The corresponding parameters of performance are shown in Table S1. Among the three QDs, CdS gives the lowest photocurrent and the highest voltage due to its wide bandgap and high CB edge. PbS offers higher current and much lower voltage than CdS. The relative high current is due to the strong ability of PbS to harvest photons. The low voltage stems from that PbS suffers a serious carrier recombination due to low CB level and high trap densities. When PbCdS-1 was applied, the highest current (11.2 mA/cm^2^) was obtained, while the voltage is larger than that of PbS. The highest current stems mainly from the high IPCE of PbCdS-1 as discussed above. In addition to an efficient photon harvesting ability, there might be other reasons that contribute to the high current and voltage. According to recent publications, Hg- or Cu-doped PbS could push the CB position to a higher energy level, which further promotes the electron injection and leads to a high current [34,49]. In another recent research work, the introduction of Cd into PbS was found to bring a similar result [42,45]. Therefore, one can confirm that the CB edge of PbCdS-1 is much higher than for PbS, which means that the introduction of Cd into PbS is favorable for the electron injection. This favorable electron injection could reduce electron recombination between PbCdS-1 and TiO_2_, which contributes to a higher voltage. Another reason might be the reduced defects by alloying Cd into PbS that may decrease trap density and restrain the recombination between TiO_2_ and QDs.

To study the effect of the proportions of Pb and Cd in the Pb*_x_*Cd_1−*x*_S QDs, two more Pb*_x_*Cd_1−*x*_S QDs—PbCdS-2 and PbCdS-3—were investigated as photosensitizers for QDSC applications. PbCdS-1, -2, and -3 were prepared with gradually decreased Pb^2+^ concentrations of precursor solution, as shown in Table S2. Inductively coupled plasma-atomic emission spectrometry (ICP-AES) was carried out to measure the three kinds of Pb*_x_*Cd_1−*x*_S QD electrodes prepared by five SILAR cycles, directly revealing the concentrations of Pb and Cd of Pb*_x_*Cd_1−*x*_S QDs listed in Table S3. Therefore, the corresponding chemical formula of PbCdS-1, -2, and -3 can be deduced as Pb_0.54_Cd_0.46_S, Pb_0.31_Cd_0.69_S, and Pb_0.24_Cd_0.76_S, respectively. Although these formulas may not be quite exact for other cycles of Pb*_x_*Cd_1−*x*_S QDs, the proportion of Pb in the Pb*_x_*Cd_1−*x*_S QDs can be confirmed to be in the order: PbCdS-1 > PbCdS-2 > PbCdS-3.

**Figure 2:**
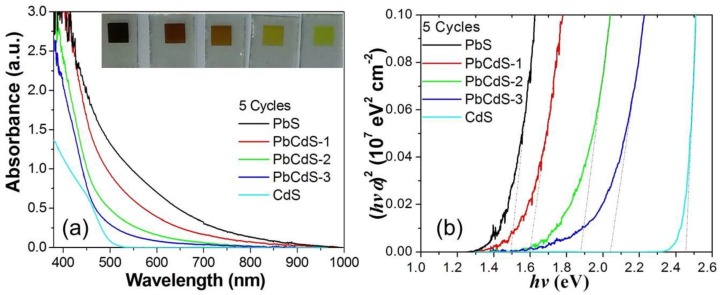
Absorption spectra (**a**) and Tauc plots (**b**) of five-SILAR-cycle PbS, PbCdS-1, -2, -3, and CdS-sensitized TiO_2_ films. The bare TiO_2_ film was used as blank in the absorption measurement. Inset images of (**a**) show the PbS, PbCdS-1, -2, -3, and CdS electrodes in turn from left to right. The doted tangent lines of the linear region of plots in (**b**) show the linear fit for the bandgap energy of QDs.

The absorption spectra of the five-SILAR-cycle QD-sensitized electrodes are shown in Figure 2a. Compared to the inset of Figure 1a, the five-SILAR-cycle CdS, PbS, and PbCdS-1 show stronger absorbance intensities and broader absorption ranges than the corresponding three-SILAR-cycle QDs. Increasing the number of cycles makes the QDs to grow bigger in size, leading to narrower bandgap and stronger and broader absorption. The effect of the proportion of Pb in the Pb*_x_*Cd_1−*x*_S QD alloys on the optical properties is illustrated by comparing the absorption spectra of PbCdS-1, PbCdS-2, and PbCdS-3. It is clearly indicated that the absorbance intensity follows the order: PbCdS-1 > PbCdS-2 > PbCdS-3. Moreover, derived from the absorption spectra, the Tauc plots of (hνα)^2^ *vs.* hν, drawn in Figure 2b, can be employed to estimate the optical bandgap related by the Tauc equation [48].

(1) $${\left(h\nu \alpha \right)}^{2}\propto (h\nu -E_{g}^{\mathrm{op}})$$

where $E_{g}^{\text{op}}$ is optical bandgap, *ν* is light frequency, and α is the absorption coefficient. Therefore, the bandgap is determined by the intersection of the base line and the tangent line.

**Figure 3:**
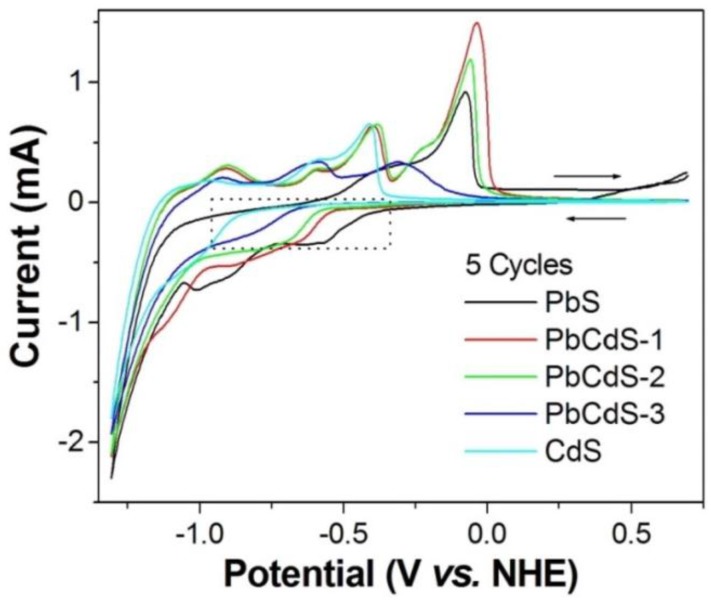
Cyclic voltammograms (CVs) of five-SILAR-cycle PbS, PbCdS-1, -2, -3, and CdS-sensitized TiO_2_ electrode. CVs were measured with the electrolyte of 0.1 M KCl in deionized water (pH 6.9), and the scan rate was 10 mV/s. The arrows indicate the scan direction. The dotted part in the CV figures is to show the onset potentials of the reduction current varied at the different electrodes.

**Scheme 1:**
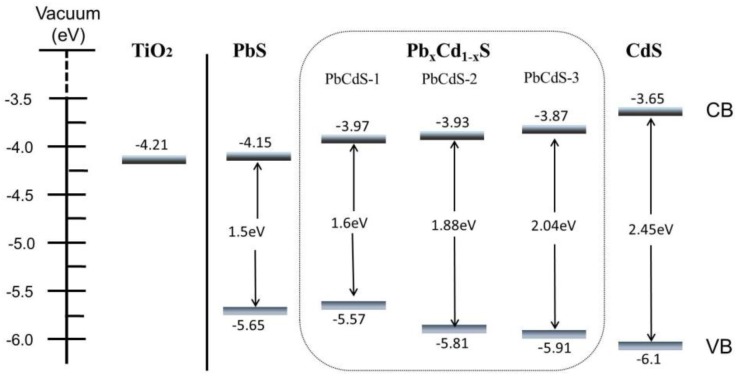
Schematic diagram of edge energy levels of the conduction band (CB) edge and valence band (VB) edge for TiO_2_ and five-SILAR-cycle PbS, CdS, and three kinds of Pb*_x_*Cd_1−*x*_S.

As discussed above, the CB energy level is an important factor affecting the performance of QDs solar cells. In this study, cyclic voltammogram (CV) was carried out in a 0.1 M KCl aqueous solution at pH 6.9, shown in Figure 3. The CB edge can be determined by the onset potential of the reduction current [50,51]. Compared to the CV performance of bare FTO and TiO_2_ electrodes (Figure S1), the onset parts of the reduction current surrounded by the dotted line should result from QDs. Therefore, combining the obtained bandgaps and CB edges, the relationship of energy levels for different QDs and TiO_2_ is shown in Scheme 1. The CB edge of TiO_2_ was −4.21 eV (*vs.* vacuum) taken from the literature [52]. The CB energy value *vs.* normal hydrogen electrode (NHE) obtained from the CV was converted to the vacuum level by the relation that 0 V *vs.* NHE is equal to −4.5 eV *vs.* vacuum [53]. Clearly, as shown in Scheme 1, the bandgap of Pb*_x_*Cd_1−*x*_S QDs is wider than for PbS and narrower than for CdS under the same SILAR cycles, and the higher proportion of Pb in Pb*_x_*Cd_1−*x*_S presents a lower bandgap. In other words, increasing the proportion of Pb leads to a higher absorbance intensity and a concomitant shift of the absorption feature towards the higher wavelength because the larger proportion of Pb decreases the bandgap of the QDs. Moreover, it is confirmed that the CB positions of Pb*_x_*Cd_1−*x*_S QDs are between PbS and CdS QDs, and PbS QDs have a low CB edge (−4.15 eV) close to the TiO_2_ CB edge. Therefore, a higher CB level causes a faster electron injection from QDs into TiO_2_.

**Figure 4:**
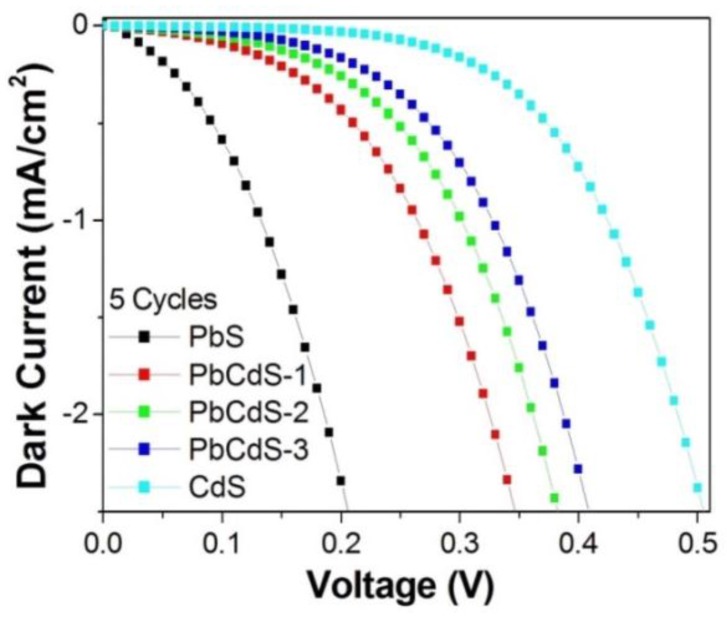
Dark I–V curves of QDSCs employing five-SILAR-cycle PbS, PbCdS-1, -2, -3, and CdS.

Generally, the major factors limiting the conversion efficiency of QDSC are the relatively slow electron transportation within the TiO_2_ film and the slow hole transfer, the main consequence of which is a large carrier loss due to the recombination between the TiO_2_ film and electrolyte [54]. Moreover, the trap states of QDs in the middle position between the TiO_2_ CB edge and the redox level of the electrolyte [3], which are generated by the interfacial effect and inner defects, can aggravate the recombination through back electron transfer via trap states. Therefore, it is vital to reduce the trap density of QDs for improving the performance of QDSCs. As discussed above, the presence of Cd in PbS may reduce the trap states. In order to verify the effect of Cd, dark I–V measurements were carried out, which is a good method to investigate the carrier recombination between TiO_2_ and electrolyte. As shown in Figure 4, the onset of dark current of the devices based on Pb*_x_*Cd_1−*x*_S occurred at a much higher bias-voltage than that of PbS, indicating that the recombination in Pb*_x_*Cd_1−*x*_S-based QDSCs is remarkably reduced. It also suggests that the increase of Cd in Pb_x_Cd_1−x_S can further reduce the recombination degree. The result proved that the utilization of Pb_x_Cd_1−x_S can effectively decrease the trap density.

**Figure 5:**
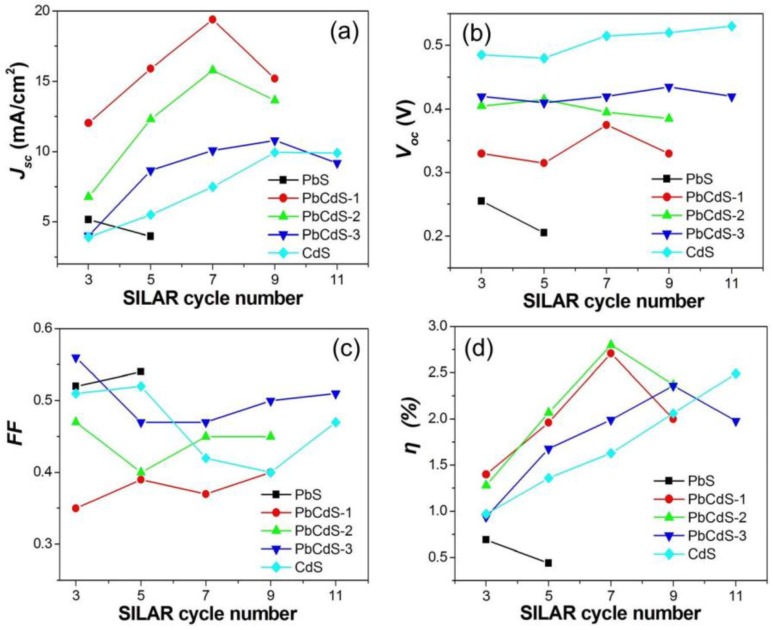
The photovoltaic parameters of QDSCs employing CdS, PbS, and three Pb*_x_*Cd_1−*x*_S QDs in terms of the number of SILAR cycles. (**a**) short-circuit current density (*J_sc_*); (**b**) open-circuit voltage (*V_oc_*); (**c**) fill factor (*FF*); (**d**) efficiency (η).

The optimization of the SILAR cycles is a key factor to boost the performance of the solar cells. Herein, we discuss the effect of SILAR cycles on the photovoltaic performance of the solar cells. The I–V curves of the solar cells of PbS, CdS, and Pb*_x_*Cd_1−*x*_S under one sun illumination are depicted in Figure S2. The photovoltaic parameters are listed in Table S4. In order to analyze the effect of the SILAR cycles, the photovoltaic parameters were presented as a function of the number of such cycles as shown in Figure 5. It was observed that the performance of the PbS solar cells decreases with the increase of SILAR cycles, probably due to the narrower bandgap and the lower CB energy level of the PbS QDs when the SILAR cycles increases after three cycles. The photocurrents of the three kinds of Pb*_x_*Cd_1−*x*_S and CdS are enhanced by increasing SILAR cycles until reaching a number of certain cycles. To further optimize the performance by increasing the cycle number, it was found that 7-cycle PbCdS-1, 7-cycle PbCdS-2, 9-cycle PbCdS-3, and 9-cycle CdS QDSCs provide the best photocurrent. Moreover, the trend of the efficiency is similar to that of the photocurrent. The comparison of the parameters between the different QD solar cells indicates that the QDs with the higher proportion of Pb show the higher short-circuit current and the lower open-circuit voltage.

**Figure 6:**
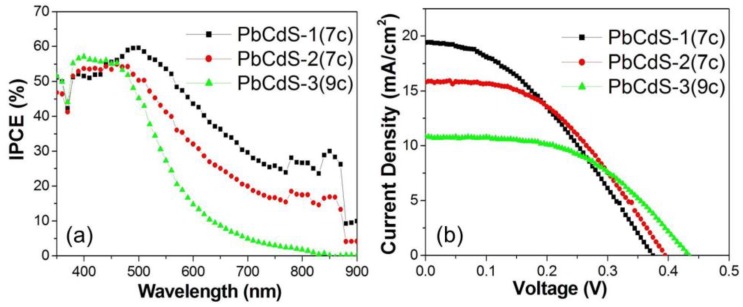
(**a**) IPCE spectra and (**b**) I–V curves of QDSCs employing PbCdS-1 (seven SILAR cycles), PbCdS-2 (seven SILAR cycles), and PbCdS-3 (nine SILAR cycles).

After analyzing the optimization of solar cell parameters, some characteristic trends can be unraveled. In particular, the proportion of Pb and Cd in the Pb*_x_*Cd_1−*x*_S alloys will affect these parameters. In order to better understand the effect, the optimized performance of the solar cells based on Pb*_x_*Cd_1−*x*_S QDs are re-illustrated and compared in Figure 6; these are the PbCdS-1 (seven SILAR cycles), PbCdS-2 (seven SILAR cycles), and PbCdS-3 (nine SILAR cycles) QDSCs. These three samples show a similar maximum IPCE close to 60%. However, a clear broadening of the IPCE features is observed for the PbCdS-1 solar cell which presents an IPCE range up to more than 900 nm, while the PbCdS-3 only reaches 800 nm. In other words, increasing the proportion of Pb (and thus decreasing the proportion of Cd) in the Pb*_x_*Cd_1−*x*_S alloys leads to a significant broadening of the IPCE features. This result is consistent with the absorption performance of the five cycles Pb_x_Cd_1−x_S. Moreover, the IPCE trend also reflects the photocurrent in the I–V curves of the QDSCs. It is clear that the photocurrent follows the trend: PbCdS-1 > PbCdS-2 > PbCdS-3, something that can be mainly attributed to the light-harvesting ability of the Pb*_x_*Cd_1−*x*_S alloys. The highest photocurrent *J_sc_* obtained by the PbCdS-1 QDSCs reaches almost 20 mA/cm^2^. However, the voltage follows the reverse trend: PbCdS-1 < PbCdS-2 < PbCdS-3. Increasing the proportion of Pb leads to a lower voltage, which is caused by the effect of a lower CB level and higher trap density, as discussed above. PbCdS-2 QDSCs exhibit the efficiency of 2.8%, referring to their moderate photocurrent of 15.8 mA/cm^2^ and voltage of 0.4 V. Therefore, controlling the ratio between Pb and Cd in the Pb*_x_*Cd_1−*x*_S QDs alloys is very important for the photovoltaic performance of the QDSCs, especially for controlling the photocurrent and voltage.

**Figure 7:**
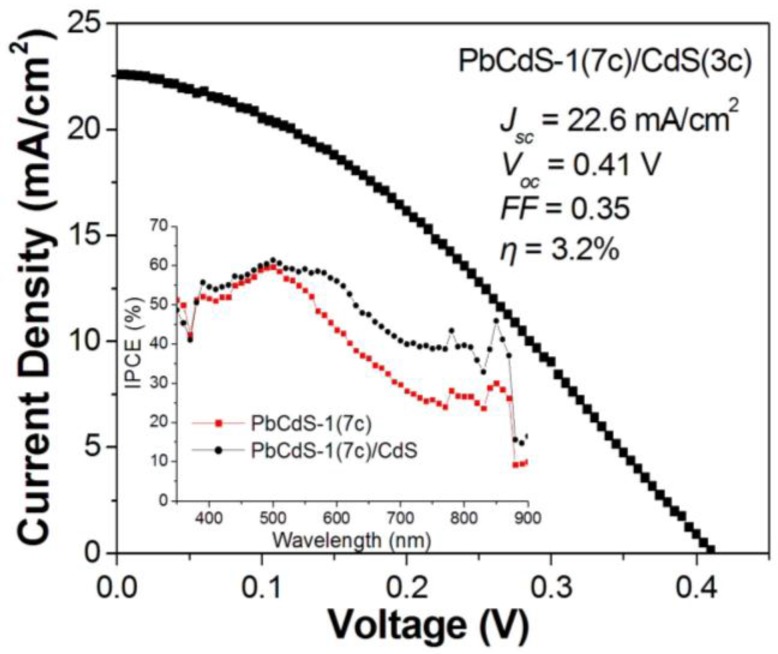
I–V curve of QDSC employing a hybrid QDs of PbCdS-1 (seven SILAR cycles) and CdS (three SILAR cycles), where PbCdS-1 was sensitized onto a TiO_2_ film, followed by subsequently depositing CdS onto PbCdS-1. Inset: IPCE spectrum of the hybrid QDs compared to the corresponding PbCdS-1 QDs.

Furthermore, we also investigated the photovoltaic performance after an extra CdS coating layer was introduced onto the PbCdS-1 QDs, shown in Figure 7. The CB edge of CdS is higher than that of Pb*_x_*Cd_1−*x*_S, which will favor the electron injection of CdS to TiO_2_ via PbCdS-1. Therefore, it could be observed that the IPCE of seven-cycle PbCdS-1 QDSCs after coating three-cycle CdS QDs improve, especially at wavelengths larger than 500 nm. In addition, CdS coating could serve as a protection layer to prevent the inner layered QDs from the corrosion of electrolyte and reduce the charge recombination [30]. This improvement gives us finally a photocurrent of 22.6 mA/cm^2^ and an efficiency of 3.2% for the QDSC.

## 3. Experimental Section

### 3.1. Preparation of the QDs-Sensitized Solar Cells

Mesoporous TiO_2_ films with a triple-layer structure, containing the compact, transparent, and scattering layer, were prepared according to the procedure reported in [55]. First, fluorine-doped tin oxide (FTO, Solaronix TCO22-7) glass was sequentially cleaned in saturated sodium hydroxide isopropanol solution, absolute ethanol, and deionized (DI) water for 15 min under ultrasonic treatment. To deposit the first layer, *i.e.*, compact layer, onto the FTO substrate, a freshly aqueous TiCl_4_ solution (40 mM) was used to treat the cleaned FTO glass for 30 min at 70 °C. Subsequently, the transparent and scattering layers were successively deposited by screen printing with TiO_2_ pastes (Solaronix, Ti-Nanoxide T/SP and R/SP), followed by sintering at 500 °C for 30 min in a muffle furnace to remove organic components. The sintered film was post-treated with an aqueous TiCl_4_ solution. The produced TiO_2_ films provided a thickness of 11 µm (7 µm for transparent layer and 4 µm for scattering layer) and a working area of 5 mm × 5 mm.

QD-sensitized TiO_2_ photoelectrodes were fabricated by growing QDs directly onto a TiO_2_ film according to the method of successive ionic layer adsorption and reaction (SILAR). In this work, PbS, CdS, and Pb*_x_*Cd_1−*x*_S QD alloys were sensitized onto TiO_2_ films to produce the photoanode. In order to sensitize these QDs, several necessary precursor solutions containing different ions should be prepared: 0.1 M Cd(NO_3_)_2_ aqueous solution as a Cd^2+^ source, 0.004 M PbCl_2_ aqueous solution as a Pb^2+^ source, and 0.1 M Na_2_S solution in methanol/DI water (1:1 by volume) as a S^2−^ source. The aqueous ionic solution of mixed Pb^2+^ and Cd^2+^ made by adding 1%–4% PbCl_2_ into 0.1 M Cd(NO_3_)_2_ solution was used as a cationic source to grow Pb*_x_*Cd_1−*x*_S QDs upon reacting with an anionic S^2−^ source. In this work, three kinds of Pb*_x_*Cd_1−*x*_S QDs, termed as PbCdS-1, PbCdS-2, and PbCdS-3, were prepared by the mixed precursor solution containing Pb^2+^ of 0.004 M, 0.002 M, and 0.001 M, respectively. The typical SILAR procedure is described by using PbS QDs as an example. The TiO_2_ film was alternately immersed into the Pb^2+^ source for 2 min and the S^2−^ source for another 2 min. The films were washed thoroughly with DI water and methanol to remove excess precursor between each immersion step. Such a single procedure is denoted as one growth cycle, and repeating the cycle to deposit more PbS onto the TiO_2_. The precursor solutions containing Pb^2+^ were replaced by a fresh identical solution for a further immersion process every two SILAR cycles. After QDs sensitization, the surface of the QDs was passivated by depositing a layer coating of ZnS in order to optimize the performance of solar cells. The ZnS coating was prepared by two SILAR cycles using a 0.1 M methanol solution of a Zn(NO_3_)_2_ as Zn^2+^ source.

The preparation process of Cu_2_S counter electrode is similar to that in the previous report [56]. Briefly, a tailored brass foil was immersed in HCl solution for 5 min at 70 °C, cleaned by DI water, and subsequently dipped into an aqueous polysulfide solution containing 1 M S and 1 M Na_2_S to generate a layer of fresh Cu_2_S onto the brass substrate. The sandwich structure of QDSCs was fabricated by assembling a QD-sensitized TiO_2_ film and Cu_2_S counter electrode with a 50-µm Surlyn film as the sealing thermoplastic material. Polysulfide electrolyte, containing Na_2_S (2 M) and S (3 M) in water/methanol (7:3 by volume), was injected to empty space between the two electrodes through a hole on the counter electrode.

### 3.2. Measurement and Equipment

UV-Vis spectra of the QD-deposited TiO_2_ films were measured by a Lambda 750 UV-Vis spectrometer (PerkinElmer, Waltham, MA, USA). The current–voltage (I–V) characteristics of the solar cell were performed by using a Keithley source-meter under AM 1.5 illumination (100 mW·cm^−2^) from a Newport 300 W solar simulator (Newport Corporation, Irvine, CA, USA). The I–V measurement setup was calibrated by using an IR-filtered silicon solar cell (Fraunhofer ISE, Freiburg, Germany). Incident photon-to-current conversion efficiencies (IPCEs) were determined using a measurement system including a 300 W xenon lamp, a 1/8 m monochromator, a Keithley source-meter, and a power meter with a 818-UV detector head. IPCEs were measured by monitoring photocurrent from low to high wavelength every 10 nm. The measured IPCE at higher wavelengths was lower than the actual value because PbS QDSCs are not stable and have slow photoelectric response. The cells with strong photoelectric response at above ~750 nm exhibit a similar fluctuation in IPCE. This should be an equipment problem, possibly resulting from the xenon lamp in the IPCE system. To weaken the light refection, a black mask (6 mm × 6 mm) was used for photovoltaic measurements. The inductively coupled plasma-atomic emission spectrometry (ICP-AES) was measured by a Perkin Elmer Model Optima 7300DV ICP AEOS (PerkinElmer, Waltham, MA, USA). Under each condition, at least three samples were prepared for the measurements in order to obtain a medium value as the final data. Cyclic voltammogram (CV) was carried out at ambient temperature with a electrochemical workstation (model 660A, CH Instruments, Austin, TX, USA) using a regular three-electrode electrochemical cell. The measured sample films were used as the working electrode, a Pt sheet as the counter electrode, and a Ag/AgCl electrode as the reference electrode. The potential conversion of reference from the Ag/AgCl electrode to normal hydrogen electrode by E (*vs.* NHE) = E (*vs.* Ag/AgCl) + 0.197 V [57].

## 4. Conclusions

This work was motivated by the fact that, while PbS quantum dots (QDs) have been successfully applied in heterojunction solar cells, showing remarkable conversion efficiency, corresponding PbS QD-sensitized solar cells (QDSCs) still have shown low device performance. One way to improve such QDSCs and utilize the high underlying quantum efficiency is alloying other metals to give a higher quality QDs. We have therefore fabricated ternary alloy Pb*_x_*Cd_1−*x*_S photosensitizers for quantum-dot-sensitized solar cells (QDSCs) by the process of successive ionic layer adsorption and reaction (SILAR). The photovoltaic performance of the QDSCs based on three kinds of Pb*_x_*Cd_1−*x*_S QDs were explored by the comparison with the corresponding PbS and CdS QDs. Firstly, we found that the three-SILAR-cycle PbCdS-1 (Pb_0.54_Cd_0.46_S) presents a much higher photocurrent compared to PbS and CdS. Then, by investigation of the absorption spectrum, cyclic voltammogram (CV), and dark I–V current of five-SILAR-cycle QDs, it is suggested that Pb*_x_*Cd_1−*x*_S QDs have a wider absorption range compared to the CdS QDs and a higher conduction band (CB) edge and reduced trap density compared to the PbS QDs. This indicates that the Pb*_x_*Cd_1−*x*_S QD alloys can overcome the shortcomings of CdS and PbS for QDSC applications. Furthermore, by comparing the PbCdS-1, PbCdS-2 (Pb_0.31_Cd_0.69_S), and PbCdS-3 (Pb_0.24_Cd_0.76_S) QDSCs, we found that the solar cells based on the Pb*_x_*Cd_1−*x*_S alloy with a higher proportion of Pb exhibit a larger photocurrent and a lower photovoltage. As a result, the PbCdS-1 solar cells present a significant short-circuit current density (*J_sc_*), up to 20 mA/cm^2^, by the optimization of the SILAR cycles. This indicates that the employment of Pb*_x_*Cd_1−*x*_S QD alloys can be a strategy to improve the photocurrent for the PbS and CdS QDSCs. Indeed, the control of the ratio of Pb:Cd in the Pb_x_Cd_1−x_S alloys is very critical for the photovoltaic performance of QDSCs based on Pb*_x_*Cd_1−*x*_S QDs. A good proportion and balance of Pb and Cd is thus required for achieving an optimum current and voltage of the solar cells. Finally, a coating layer of CdS deposited onto Pb*_x_*Cd_1−*x*_S photoelectrode gives enhancements in the photocurrent to 22.6 mA/cm^2^ and in the efficiency to 3.2%. It is reasonable to anticipate the remarkable enhancement of QDSCs by improving QD quality with less or even no defects and developing more effective passivation materials.

## Supplementary Materials

The following are available online at http://www.mdpi.com/2079-4991/6/6/97/s1. Table S1: The photovoltaic parameters of QDSCs employing three SILAR cycles of CdS, PbS, and PbCdS-1 as photosensitizers. Table S2: The concentrations of precursor solutions of cationic sources used in this work. Table S3: The concentrations of Pb and Cd in Pb*_x_*Cd_1−*x*_S QDs were assayed by inductively coupled plasma-atomic emission spectrometry (ICP-AES). Table S4: The photovoltaic parameters of QDSCs depending on the number of SILAR cycles, employing CdS, PbS, and Pb*_x_*Cd_1−*x*_S as photosensitizers; short-circuit current (*J_sc_*), open-circuit voltage (*V_oc_*), fill factor (*FF*), efficiency (η). Figure S1: Cyclic voltammograms of the bare FTO glass and TiO_2_ electrode measured under the same condition with Figure 3. Figure S2: I–V curves of QDSCs depending on the number of SILAR cycles, employing (a) CdS, (b) PbS, (c) PbCdS-1, (d) PbCdS-2, (e) PbCdS-3 as photosensitizers.
