# Supplementary material for: Improving the Photocurrent in Quantum-Dot-Sensitized Solar Cells by Employing Alloy PbxCd1−xS Quantum Dots as Photosensitizers
Source: Nanomaterials (Basel). 2016 May 27;6(6):97. doi: 10.3390/nano6060097 (PMC5302620; doi:10.3390/nano6060097)
Supplement: Supplementary file 1 [file nanomaterials-06-00097-s001.pdf]

Supplementary Materials: Improved Photocurrent in Quantum Dot Sensitized Solar Cells by Employing Alloy $Pb_xCd_{1-x}S$ QDs as Photosensitizers

Chunze Yuan, Lin Li, Jing Huang, Zhijun Ning, Licheng Sun and Hans Ågren

Table S1. The photovoltaic parameters of quantum-dot-sensitized solar cells (QDSCs) employing 3 successive ionic-layer adsorption and reaction (SILAR) cycles of CdS, PbS, and PbCdS-1 as photosensitizers.

Photosensitizer	J_{sc} (mA/cm^2)	V_{oc} (V)	*FF*	η (%)
CdS(3c)	3.88	0.48	0.50	0.93
PbS(3c)	5.64	0.24	0.51	0.69
PbCdS-1(3c)	11.22	0.28	0.49	1.54

Table S2. The concentrations of precursor solutions of cationic sources used in this work.

QDs	Cd^{2+} (M)	Pb^{2+} (M)
PbS	0	0.004
PbCdS-1	0.1	0.004
PbCdS-2	0.1	0.002
PbCdS-3	0.1	0.001
CdS	0.1	0

Table S3. The concentrations of Pb and Cd in $Pb_xCd_{1-x}S$ QDs were assayed by Inductively Coupled Plasma Atomic Emission Spectrometry (ICP-AES).

Concentrations	PbCdS-1	PbCdS-2	PbCdS-3
Concentration Cd (μg/mL)	29.03	39.14	37.69
Concentration Pb (μg/mL)	61.95	32.25	21.49
Calculated Chemical formula	$Pb_{0.54}Cd_{0.46}S$	$Pb_{0.31}Cd_{0.69}S$	$Pb_{0.24}Cd_{0.76}S$

Table S4. The photovoltaic parameters of QDSCs depending on the number of SILAR cycles, employing CdS, PbS, and $Pb_xCd_{1-x}S$ as photosensitizers; short-circuit current: J_{sc}, open-circuit voltage: V_{oc}, fill factor: *FF*, efficiency: η.

Photosensitizer	J_{sc} (mA/cm^2)	V_{oc} (V)	*FF*	η (%)
PbS(3c)	5.16	0.255	0.52	0.69
PbS(5c)	3.97	0.205	0.54	0.44
PbCdS-1(3c)	12.04	0.330	0.35	1.40
PbCdS-1(5c)	15.92	0.315	0.39	1.96
PbCdS-1(7c)	19.40	0.375	0.37	2.71
PbCdS-1(9c)	15.22	0.330	0.40	2.00
PbCdS-2(3c)	6.77	0.405	0.47	1.28
PbCdS-2(5c)	12.32	0.415	0.40	2.07
PbCdS-2(7c)	15.80	0.395	0.45	2.80
PbCdS-2(9c)	13.66	0.385	0.45	2.37
PbCdS-3(3c)	4.02	0.420	0.56	0.94
PbCdS-3(5c)	8.66	0.410	0.47	1.68
PbCdS-3(7c)	10.09	0.420	0.47	1.99
PbCdS-3(9c)	10.81	0.435	0.50	2.36
PbCdS-3(11c)	9.19	0.42	0.51	1.98

Table S4. *Cont.*

Photosensitizer	J_{sc} (mA/cm²)	V_{oc} (V)	*FF*	η (%)
CdS(3c)	3.93	0.485	0.51	0.97
CdS(5c)	5.50	0.480	0.52	1.36
CdS(7c)	7.50	0.515	0.42	1.63
CdS(9c)	9.94	0.52	0.40	2.06
CdS(11c)	9.92	0.53	0.47	2.49

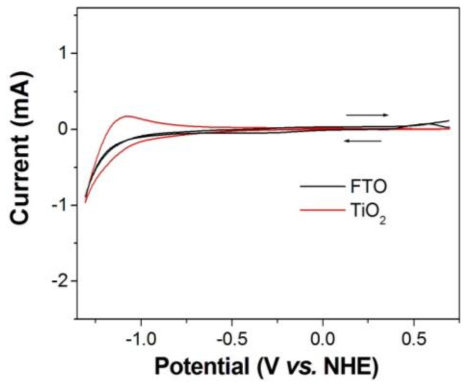

Figure S1. Cyclic voltammograms of the bare fluorine-doped tin oxide (FTO) glass and TiO_2 electrode measured under the same condition with Figure 3.

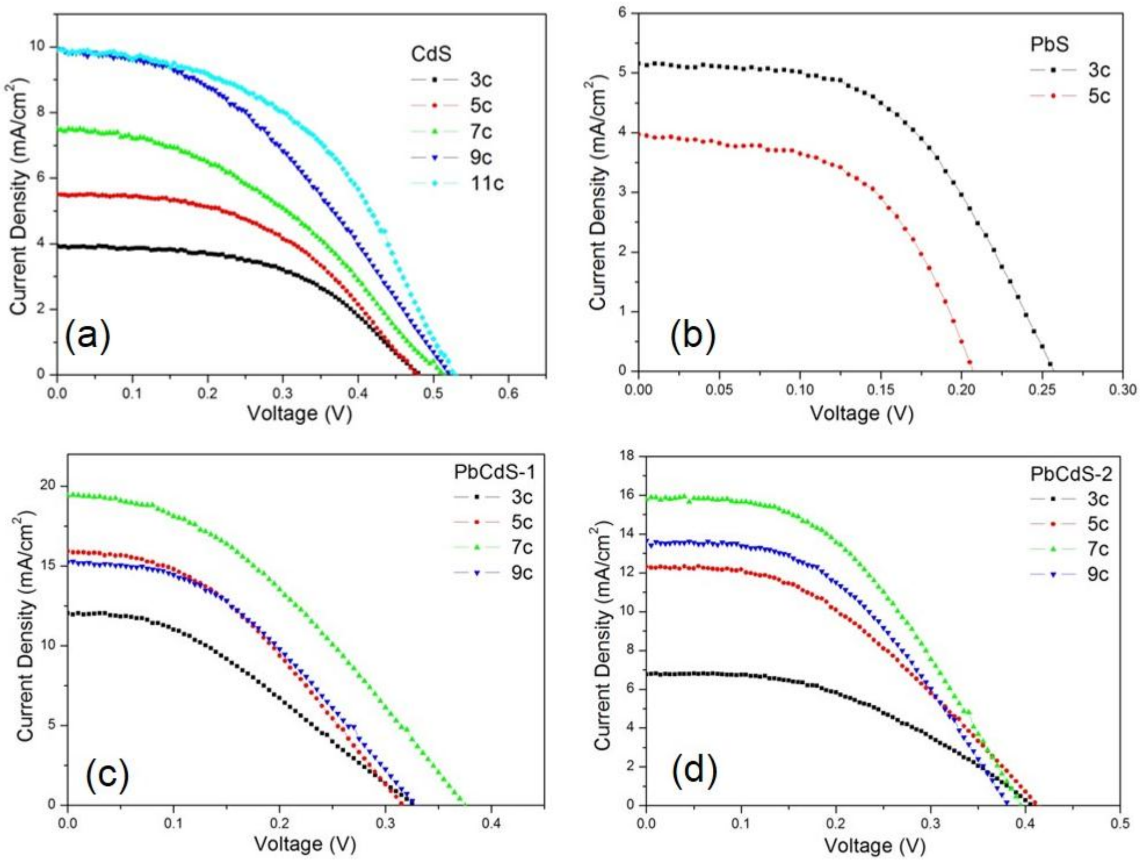

Figure S2. *Cont.*

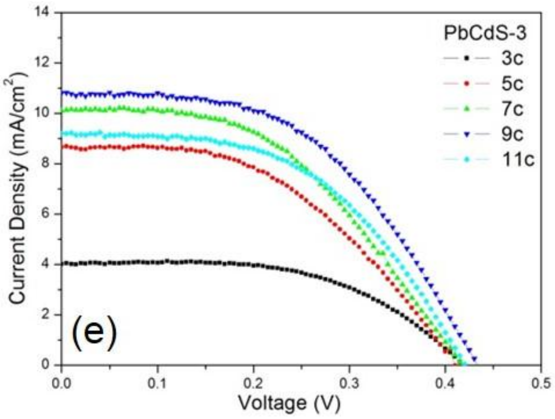

Figure S2. I–V curses of QDSCs depending on the number of SILAR cycles, employing (**a**) CdS; (**b**) PbS; (**c**) PbCdS-1; (**d**) PbCdS-2; (**e**) PbCdS-3 as photosensitizer.

© 2016 by the authors; licensee MDPI, Basel, Switzerland. This article is an open access article distributed under the terms and conditions of the Creative Commons by Attribution (CC-BY) license (http://creativecommons.org/licenses/by/4.0/).
